# Diagnostic Value of Biomarkers in the Diagnosis of Cholangiocarcinoma and Its Benign Mimickers 

**DOI:** 10.30699/ijp.2025.2029980.3307

**Published:** 2025-03-10

**Authors:** Elmira Zolfeghari Khiavy, Nasser Rakhshani, Hamid Rezvani, Behnaz Bouzari, Hossein Ajdarkosh, Mahshid Panahi, Hemin Ashayeri, Seyed Mir Yaghub Musaviani, Soheil Khalili, Ali Basi, Mohammad Hadi Karbalaie Niya

**Affiliations:** 1 *Department of Pathology, School of Medicine, Iran University of Medical Sciences, Tehran, Iran*; 2 *Gastrointestinal and Liver Diseases Research Center, Iran University of Medical Sciences, Tehran, Iran*; 3 *Department of Adult Hematology Oncology, School of Medicine, Shahid Beheshti University of Medical Sciences, Tehran, Iran*; 4 *Islamic Azad University, Tehran Branch, Tehran, Iran*; 5 *Department of Hematology Oncology, Iran University of Medical Sciences, Tehran, Iran*; 6 *Department of Virology, School of Medicine, Iran University of Medical Sciences, Tehran, Iran*

**Keywords:** Biomarker, Cholangiocarcinoma, Immunohistochemistry

## Abstract

**Background & Objective:**

Cholangiocarcinoma (CCA) is a malignancy that accounts for approximately 3% gastrointestinal cancer. The aim of this study was to evaluate and compare the diagnostic value of CD56, SMAD4, CEA, and p53 biomarkers in diagnosing cholangiocarcinoma and its benign mimickers.

**Methods:**

This retrospective cross-sectional study was conducted on 54 CCA specimens and 27 non-cancerous pancreatobiliary tissue samples diagnosed between 2018 and 2022. All specimens were evaluated using immunohistochemistry (IHC) staining for CD56, SMAD4, CEA, and p53 expression. The cut-off value of each marker was obtained from the respective kit instructions and previous studies. The results were analyzed using SPSS version 26. A significance level of less than 0.05 was considered statistically significant.

**Results:**

Of the 81 specimens, the mean age in the case and control groups was 57.33 ± 11.99 and 44.7 ± 16.69 years, respectively, and 51 (63%) samples were obtained from male patients. We found that 39.5% had grade III p53 expression, 13.5% had grade II p53 expression, 41.9% had grade III CEA expression, and 9.8% had grade II CEA expression. Additionally, 17.3% were positive for CD56 expression, and 7.4% showed SMAD4 loss. There were significant associations between the expression of CEA (79.6%) and p53 (74%) in the CCA group (p-value < 0.05). However, SMAD4 loss and CD56 expression were not statistically significant.

**Conclusion:**

Expression of CEA and p53 based on IHC staining is associated with the occurrence of CCA. However, SMAD4 and CD56 were not significantly associated with CCA. Further survival analysis and sensitivity and specificity assessments are needed to obtain more comprehensive results.

## Introduction

Cholangiocarcinoma (CCA) is a group of invasive malignancies that arise from different parts of the bile duct and within the liver parenchyma (1). It is the second most common primary liver malignancy after hepatocellular carcinoma (HCC), accounting for approximately 15% of primary liver cancers and 3% of all gastrointestinal malignancies. Although considered a rare cancer, the incidence and mortality of CCA have been steadily increasing worldwide over the past several decades (2–6).

CCA exhibits features of cholangiocyte differentiation and is likely derived from the epithelial cells lining the bile ducts, known as cholangiocytes. However, depending on the tumor location and underlying liver pathology, it may also originate from hepatocytes or peribiliary glands (1).

Based on the anatomic site of origin, CCAs are classified into intrahepatic (iCCA), perihilar (pCCA), and distal CCA (dCCA), which differ in etiology, risk factors, prognosis, as well as clinical and therapeutic management. iCCA arises within the liver parenchyma proximal to the secondary biliary ducts; pCCA is located between the secondary biliary ducts and the insertion site of the cystic duct into the common bile duct; and dCCA occurs distal to the cystic duct insertion, involving the common bile duct (1). These three subtypes show differences in causative factors, clinical behavior, treatment approaches, and outcomes (7).

The peak incidence of CCA typically occurs between the fifth and seventh decades of life, with a slight male predominance observed in iCCA cases (7). The prognosis remains poor due to the asymptomatic nature of early-stage disease and limited treatment options (8). Because CCA progresses slowly, early diagnosis is challenging, and most patients present with advanced or metastatic disease, limiting the effectiveness of therapeutic interventions (7, 8).

A negative margin resection remains the most critical factor influencing survival and prognosis. Unfortunately, a considerable proportion of patients present with locally advanced, unresectable tumors (7, 9). Given the aggressive nature and subtle clinical features of CCA, early detection is crucial for improving outcomes through expanded therapeutic options and for reducing the economic burden associated with treating advanced disease (8). The identification of specific biomarkers associated with early stages of CCA could offer a promising strategy for improving prognosis and timing of treatment (8).

Diagnosing CCA often requires extensive pathological evaluation, as its clinical, biochemical, and radiologic features are nonspecific and poorly recognized. The histopathological diagnosis of CCA remains a significant diagnostic challenge (10). Differentiating CCA from metastatic adenocarcinomas, particularly gastric and pancreatic adenocarcinomas, is particularly difficult due to morphologic and immunophenotypic overlap, making diagnosis based on morphology alone insufficient (11).

To overcome these limitations, a broad spectrum of biomarkers in tissue, serum, and other body fluids has been explored for the diagnosis and differentiation of CCA (8). The availability of reliable biomarkers can enable early detection and guide the selection of appropriate treatment strategies (8). However, variability in diagnostic performance across studies may result from differences in instrumentation, settings, and operator experience (8).

Previous research has focused on identifying biomarkers that assist in the diagnosis and understanding the pathogenesis of CCA (8, 12). In recent years, there has been increasing interest in biomarkers for diagnosing challenging cases, including those where conventional tests are inconclusive (13). Although limited data exist regarding gene-based diagnostics for CCA, some studies have identified TP53 and SMAD4 as commonly mutated genes in affected patients (14). Additionally, aberrant expression of p53, CEA, and CD56 has been observed in CCA (15–17).

In this study, we aimed to compare the expression levels of CD56, SMAD4, CEA, and p53 as potential diagnostic markers for cholangiocarcinoma.

## Materials and Methods

This cross-sectional study was conducted on specimens of patients diagnosed with CCA in a referral hospital affiliated to Iran University of Medical Sciences, Tehran, Iran, from April 2018 to April 2022. The inclusion criteria were confirmed diagnosis of CCA based on pathology, having access to pathology specimens, and having proper quality and quantity of specimens for immunohistochemistry (IHC) staining. The exclusion criteria were a lack of demographic or pathologic data in the patient’s file and not confirmed CCA.

All pathology specimens of CCA patients (cases, n = 54) and normal tissue (non-cancerous pancreatobiliary tissue) (control group, n = 27) were extracted from the pathology repository. All specimens were evaluated using immunohistochemistry (IHC) staining to assess the expression of CD56, SMAD, CEA, and p53 biomarkers. All procedures were performed using the kit’s protocol. Two blinded pathologists did the interpretations. The mean interpretation score was calculated for each evaluated specimen. Demographic and pathologic data of patients were recorded on a researcher-developed checklist based on patient files.

For the assessment of p53 expression based on IHC, the kit from GenomeMe (Canada) (clone code IHC053) was obtained. A p53-positive result was defined as staining in >10% of tumor cells with moderate to strong (++) or higher staining intensity (18, 19). The CEA kit from GenomeMe (Canada) (clone code IHC543) was performed. A CEA-positive result was defined as staining intensity in >10% of cells with weak to moderate staining (20, 21).

The DPC4 (SMAD4) (B-8) Mouse Monoclonal Antibody kit (Zeta Corporation, Canada) was used. Smad4 results were reported as intact expression (positive) when the nuclear staining or cytoplasmic and nuclear staining of tumor cells was observed in the target tissue, and loss of expression (negative) when we observed the lack of staining of the tumor cell or its nuclear and cytoplasmic staining (22).

The CD56 (123C3.D5) Mouse Monoclonal Antibody kit (Zeta Corporation, Canada) was used for the experiment. CD56 was defined as positive for membranous expression with or without cytoplasmic staining when it was seen in 10% or more of neoplastic cells (23).

Staining intensity (grade) ranged from 0 to 3 points, corresponding to: negative staining (IHC grade score 0); weak staining (IHC grade score 1); moderate staining (IHC grade score 2); and strong staining (IHC grade score 3) (24).

### Statistical Analysis

All results are presented as mean ± standard deviation or frequency (%). The relationship of qualitative variables with the outcome was calculated using the chi-square and Fisher's exact tests, and the relationship of quantitative variables with the outcome was calculated using the Mann-Whitney test. All analyses were performed using SPSSv.26 software. Statistical significance was set at *P* < 0.05.

## Results

A total of 81 patients were included in this study. Fifty-four patients (66.7%) with cholangiocarcinoma (CCA) and 27 patients (33.3%) were in the control group. Thirty (37%) samples were from women and 51 (63%) samples were from men. The mean age of all patients was 53.12 ± 14.88 years (n = 81). The mean age in the CCA group and the control group were 57.33 ± 11.99 and 44.7 ± 16.69, respectively. Demographic and basic pathologic data are shown in Table 1.

**Fig. 1:**
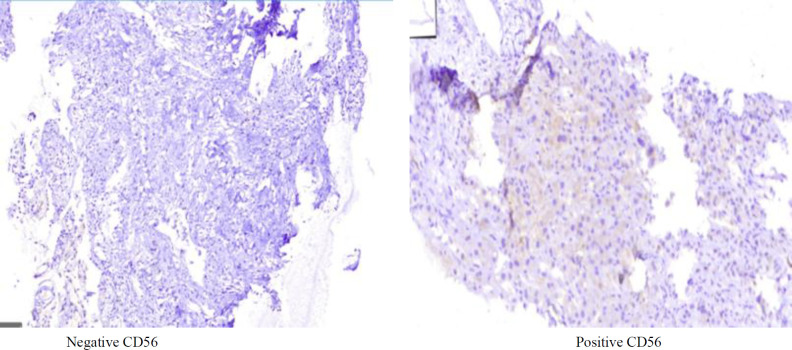
Positive and negative CD56 staining

CD56 expression was seen more frequently in cases (n = 8, 14.8%); however, this difference was not significant (*P* = 0.534) by chi-square test (Table 1). Figure 1 shows negative and positive CD56 IHC staining.

**Fig. 2:**
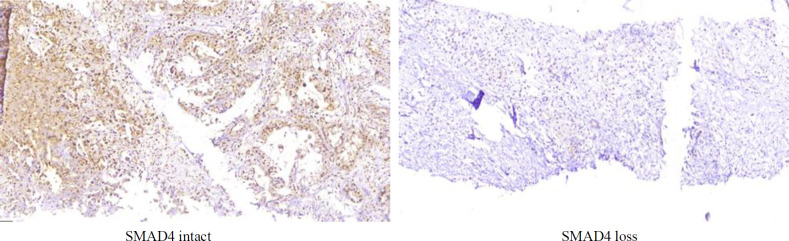
SMAD4 loss and intact features

SMAD4 loss was detected in 6 (7.4%) of the 54 CCA samples. An abnormality associated with SMAD loss, based on the tumor site, loss of SMAD4 was seen in the liver mass and common bile duct. SMAD4 IHC staining was intact in the other tumor sites and control groups. There was no significant difference between CCA and the control group related to SMAD4 results (*P* = 0.1) (Table 1). Figure 2 shows SMAD4 staining features.

**Fig. 3:**
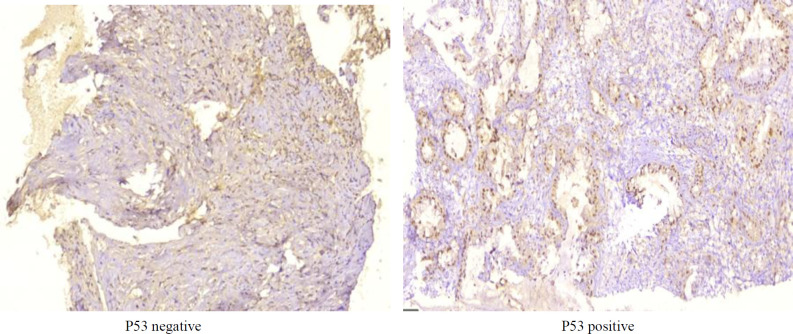
Features of P53 in the specimens

Evaluation of p53 expression revealed that 74% (n = 40) of CCA group were p53 positive, while all samples from the control group were negative. The difference between CCA and control group p53 expression was significant (*P* < 0.001). The p53 expression results based on the site of tumor are demonstrated in Table 2. Also, p53 expression was significantly higher in most CCA cases (*P* < 0.001) (Table 1). Figure 3 shows the p53 staining features.

By the CEA analysis, 79.6% (n = 43) of CCA cases and 11.1% of samples from the control group were found positive. This difference was significant (*P* = 0.002) (Table 2). Interestingly, CEA expression grades were higher in most CCA samples (*P* < 0.001) (Table 1).

Additionally, the mean of positive cells in the specimens in both p53 and CEA were different significantly (Table 3).

**Table 1:** Demographic-related and basic pathologic data of specimens

Variables	Subgroups	CCA group Number (%)	Control group Number (%)	Total per subgroup Number (%)	p-Value
Gender	Female	19 (63.3)	11 (36.7)	30 (37)	0.625 *
	Male	35 (68.6)	16 (31.4)	51 (63)	
Age (year)	18-64	36 (62.0)	22 (38.0)	58 (71.6)	0.163 *
	More than 64	18 (78.2)	5 (21.8)	23 (28.4)	
Site of tumor	Pancreatic head	2 (100)	0 (0)	2 (2.4)	<0.001 **
	Liver mass	4 (100)	0 (0)	4 (4.9)	
	CBD	40 (100)	0 (0)	40 (49.3)	
	None	8 (29.6)	27 (70.4)	35 (43.2)	
Tissue status	Pancreatobiliary carcinoma	54 (100)	0 (0)	54 (66.7)	-
	Normal tissue around tumor	0 (0)	27 (100)	27 (33.3)	
p53 expression grade	Grade 0	5 (15.7)	27 (84.3)	32 (39.5)	<0.001 **
	Grade 1	6 (100)	0 (0)	6 (7.4)	
	Grade 2	11 (100)	0 (0)	11 (13.5)	
	Grade 3	32 (100)	0 (0)	32 (39.5)	
CEA expression grade	Grade 0	11 (31.4)	24 (68.6)	35 (43.2)	<0.001 **
	Grade 1	3 (75.0)	1 (25.0)	4 (4.9)	
	Grade 2	6 (75.0)	2 (25.0)	8 (9.8)	
	Grade 3	34 (100)	0 (0)	34 (41.9)	
CD56	Positive	8 (57.1)	6 (42.9)	14 (17.3)	0.534 *
	Negative	46 (68.6)	21 (31.4)	67 (82.7)	
SMAD4	Intact	48 (64.0)	27 (36)	75 (92.6)	0.1 *
	Loss	6 (100)	0 (0)	6 (7.4)	

* Chi-square test; **Fisher's exact test.

Abbreviations: CCA: Cholangiocarcinoma; CEA: carcinoembryonic antigen; SMAD4: SMAD Family Member 4; CBD: common bile duct.

**Table 2:** The results of the p53 and CEA expression in the two groups

	CCA group				Control group			p-value *
	Negative N (%)	Positive N (%)	Total N (%)	Negative N (%)		Positive N (%)	Total N (%)	
p53 Positive	14 (26)	40 (74)	54 (100)	27 (100)		0 (0)	27 (100)	<0.001
CEA Positive	11 (20.4)	43 (79.6)	54 (100)	24 (88.9)		3 (11.1)	27 (100)	0.002

^*^Chi-square test was used.

**Table 3:** The percentage of positive cells in the p53 and CEA analysis in our studied specimens

	CCA group	Control group	Total	p**–**Value*
	Mean ± SD	Mean ± SD	Mean ± SD	
p53 Positive cells%	62.02±32.2	0.19±0.96	41.41±39.34	**<0.001**
CEA Positive cell%	54.87±39.2	4.44±11.21	38.06±40.38	**<0.001**

**Fig. 4:**
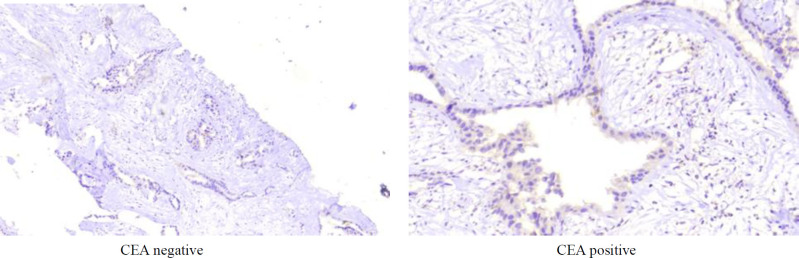
Features of CEA in the specimens

## Discussion

In the current study, we assessed 54 CCA specimens and compared the results of the IHCs with the results of normal tissue (n = 27). We found that the mean age of CCA cases and controls was 57.33 ± 11.99 and 44.7 ± 16.69, respectively, and the common bile duct (CBD) was the most common affected site. In terms of marker expression, we found: 39.5% grade III p53 expression; 13.5% grade II p53 expression; 41.9% grade III CEA expression; 9.8% grade II CEA expression; 14.8% CD56 expression; and 7.4% SMAD4 loss. The percentages of p53 positive cells and CEA positive cells were significantly higher than normal cells. Also, p53 and CEA expression were significantly different in studied groups (*P* < 0.05).

Some studies have shown that CCA commonly involves patients between the ages of 50 to 70 years and the incidence increases with increasing age (7, 25, 26). In our study, the mean age of our CCA patients was 57.33 ± 11.99 years, which was compatible with the results of previous studies. According to the study by Brindley et al (1) and Gad et al (27), the prevalence of CCA is slightly more common in males, findings that are related to higher prevalence of the primary sclerosing cholangitis in males. However, this finding was not statistically significant, possibly due to limited sample size (*P* > 0.05).

Our sample size was rather high compared with other studies of Wakizaka et al, (28) (n = 25), Kang et al. (29) (n = 42), Gütgemann et al. (30) (n = 32) and Puetkasichonpasutha et al (n = 30) (31). Previous studies have shown that serum levels of non-specific tumor biomarkers, such as carbohydrate antigen 9-19 (CA19-9), are currently measured to aid in the diagnosis of CCA, but because of the low sensitivity and specificity, especially in early stages of the disease, they are not reliable (32, 33). Several biomarkers investigated from blood samples or bile but in combination with tissue evaluation, their sensitivity and specificity for diagnosis and prognosis elevated. The serum CA19-9 and CEA could provide wide ranges of sensitivity (47.2–98.2%) and specificity (89.7–100%) for CCA compared to that in the other patients. On the other hand, tissue evaluation for CEA diagnostic sensitivity in CCA cases in combination with other biomarkers ranged from 53% to 83% (8, 34, 35). As CEA is a cell membrane glycoprotein, its expression differs between normal tissues and malignant cells, making it a beneficial biomarker assessed in the diagnosis of CCA and also for treatment monitoring or determining recurrence (36).

So, our study had a limitation to assess the serum or bile levels of CEA like other markers due to the retrospective setting and restricted data of the cases in the repository and we were unable to define the sensitivity and specificity of these biomarkers for further use. Also, our purpose was different. Meanwhile, tissue evaluation of CEA expression in our cases showed the statistically significant result compared with the control group (79.6% vs. 11.1%, respectively) (*P* = 0.002). CEA could be described as a diagnostic biomarker for CCA based on our study results.

Studies have shown that wild-type p53 has a short half-life, which makes it unable to be detected by IHC staining within the normal cells, while the mutant forms of p53 persists within the CCA tumor cells and they are more easily detectable by such diagnostic methods (31). In the current study, p53 expression was high in patients with CCA and more tumor cases were found with higher grades. On the other hand, in the control group showed negative result for p53 expression. Also, p53 is one of the key regulators of entering the G1 to S phase of cell division, and p53 alteration could lead to irregular cell growth and increased risk of tumor formation (36, 37). So, it is common to find high p53 expression levels in tumor specimens. Based on our finding, we found that 74% of CCA cases expressed p53 significantly, which led us to assume it could be considered a diagnostic marker for CCA in suspected tumor tissue cases.

In the study by Kang et al (29), the changes in both G1 to S cell cycle arrest and TGF-β/SMAD pathways were investigated by immunohistochemical staining for Rb, cyclin D1, p27, p16, p53, and SMAD4/Dpc4 in 42 intrahepatic cholangiocarcinomas (iCCA). Abnormal nuclear expression of p53 was observed in 15 (35.7%) cases. SMAD4 loss was observed in 19 (45.2%). Abnormality in the pTNM pathway was positively associated with TGF-β/SMAD loss. Abnormal expression of p53 accompanying biliary dysplasia was reported in 7 out of 13 dysplastic lesions. Altered TGF-β/SMAD pathway is a main event in cholangiocarcinogenesis (29, 36). The study by Puetkasichonpasutha et al (31) reported elevation of p53 protein level in 77% of CCA cases (n = 30).

So, we evaluated the expression levels of CEA, SMAD4, p53, and CD56 in the CCA specimens. We found that the expression levels of p53 and CEA were significantly higher in CCA cases, but CD56 and SMAD4 loss were not different between CCA and non-CCA specimens. These findings demonstrated that p53 and CEA may serve as diagnostic biomarkers to CCA.

In the current study, it was found that SMAD4 loss was observed in 11.1% of CCAs; however, this difference was not statistically significant. The result of our study was different from the study by Kang et al. Also, we found p53 expression two times higher than the study by Kang et al (74% vs. 35.7%) and was statistically significant, a finding similar to that of Puetkasichonpasutha et al. These differences may be due to differences in populations, geographical and environmental, or genetic factors. Our study was conducted on 54 CCA and 27 control specimens, a larger sample size than that in the study by Kang et al.’s study (n = 42 iCCA cases) (29).

In a study by Wakizaka et al. (28), CD56 was negative in all subtypes of CCA. No relationship between CD56 expression and the prognosis of CCA was found. However, their limited sample size may affect the results (n = 25). Similarly, we observed CD56 was negative in most of the CCA specimens. Our findings are consistent with the findings of Wakizaka et al., although our study group was larger (54 vs. 25). In the study of Gütgemann et al. (30), 98 samples of bile duct tumors were examined for CD56 expression by IHC staining, and they found positive in 4 samples out of 32 cholangiocarcinoma (12.5%). In this study, 12 samples out of 17 bile duct adenocarcinomas were positive for CD56 (70.5%). CD56 can help to differentiate non-neoplastic proliferation and neoplasia. No association between CD56 expression and CCA was found in the current study. These findings were similar to those in the study by Gutgemann et al.

We did not assess other types of bile duct cancers like Gütgemann et al.; however, our cases were higher than the number of cases in their study (54 vs. 32). In the study of Chiang et al. (38), on Epstein–Barr virus (EBV)-associated lymphoepithelioma-like cholangiocarcinoma (EBV-LELCC) cases as a rare type of iCCA, found that the tumor immune microenvironment (TIME) demonstrates increased Th1 cells, NK CD56 cells, and M1 macrophages, which are associated with longer survival.

Rare studies compared normal pancreatobiliary tissue with CCA tissue cases for biomarker study in which we used these groups for more comprehensive results.

As we mentioned before, we have some limitations in our study. The sensitivity and specificity study of the biomarkers could make more comprehensive results but as a retrospective study we could not assess them due to restriction for study group sampling and missing data in the repository. Finding adequate samples from biopsy specimens or limited resected cases tissue to use for extra testing restricted us from enrolling a greater sample size; however, as mentioned, our study group was comparable with other studies (28-31). Survival analysis was another limitation of our study, which could reflect our findings outcome on patients, but in a prospective cohort setting, we might obtain such a result. 

## Conclusion

In conclusion, the use of immunohistochemical tests as a cheap and available technique has great potential to diagnose various diseases, especially in cancers. The four biomarkers panel consist of CD56, p53, SMAD4, and CEA assessed by the present study but the results showed significant relationships just between the expression of p53 (74% positivity rate, *P* < 0.001) and CEA (79.6% positivity rate, *P* = 0.002) with CCA. There were no significant relationship regarding CD56 and SMAD4 loss. p53 and CEA combination in IHC testing for CCA cases have diagnostic value; however, sensitivity and specificity values need more studies by prospective setting. 

## Data Availability

Data are available upon reasonable request from the corresponding author.
